# Supplementary material for: Non-Invasive Evaluation of Intradiscal Deformation during Axial Loading of the Spine Using Deformation-Field Magnetic Resonance Imaging: A Potential Tool for Micro-Instability Measurements
Source: J Clin Med. 2022 Aug 10;11(16):4665. doi: 10.3390/jcm11164665 (PMC9410209; doi:10.3390/jcm11164665)
Supplement: Supplementary file 1 [file jcm-11-04665-s001.zip › Suplementary Table S1 .pdf]

Table S1. Image registration parameters.

	Rigid registration	Non-rigid registration
FixedInternalImagePixelType	Float	Float
MovingInternalImagePixelType	Float	Float
UseDirectionCosines	True	True
Registration	MultiResolutionRegistration	MultiResolutionRegistration
Interpolator	BSplineInterpolator	BSplineInterpolator
ResampleInterpolator	FinalBSplineInterpolator	FinalBSplineInterpolator
Resampler	DefaultResampler	DefaultResampler
FixedImagePyramid	FixedRecursiveImagePyramid	FixedRecursiveImagePyramid
MovingImagePyramid	MovingRecursiveImagePyramid	MovingRecursiveImagePyramid
Optimizer	AdaptiveStochasticGradientDescent	AdaptiveStochasticGradientDescent
Transform	EulerTransform	BSplineTransform
Metric	AdvancedMattesMutualInformation	AdvancedMattesMutualInformation
AutomaticScalesEstimation	True	-
AutomaticTransformInitialization	True	-
FinalGridSpacingInPhysicalUnits	-	16
HowToCombineTransforms	Compose	Compose
NumberOfHistogramBins	32	32
ErodeMask	False	False
NumberOfResolutions	4	4
MaximumNumberOfIterations	250	500
NumberOfSpatialSamples	2048	2048
NewSamplesEveryIteration	True	True
ImageSampler	Random	Random

BSplineInterpolationOrder	1	1
FinalBSplineInterpolationOrder	3	3
DefaultPixelValue	0	0
WriteResultImage	True	True
ResultImagePixelType	Short	Short
ResultImageFormat	mhd	mhd
