# Supplementary material for: Non-Invasive Evaluation of Intradiscal Deformation during Axial Loading of the Spine Using Deformation-Field Magnetic Resonance Imaging: A Potential Tool for Micro-Instability Measurements
Source: J Clin Med. 2022 Aug 10;11(16):4665. doi: 10.3390/jcm11164665 (PMC9410209; doi:10.3390/jcm11164665)
Supplement: Supplementary file 1 [file jcm-11-04665-s001.zip › Suplementary Table S2.pdf]

Table S2. Linear regression results.

L1-L2

Univariable

β (p) R^2

	Pfirrmann	IVD Height	Tilt angle	Disc angle
Region 1	0,021 (0,464) 0,016	-0,009 (0,255) 0,038	-0,005 (0,367) 0,024	-0,009 (0,111) 0,073
Region 2	0,025 (0,335) 0,027	-0,011 (0,109) 0,074	-0,004 (0,455) 0,016	-0,004 (0,409) 0,020
Region 3	0,026 (0,288) 0,033	-0,011 (0,085) 0,085	-0,002 (0,623) 0,007	-0,00005 (0,992) 0,000003
Region4	0,023 (0,313) 0,030	-0,012 (0,040) 0,118	-0,002 (0,720) 0,004	0,002 (0,619) 0,007
Region 5	0,020 (0,346) 0,026	-0,015 (0,006) 0,201	-0,002 (0,606) 0,008	0,002 (0,623) 0,007
Mean	0,031 (0,143) 0,062	-0,013 (0,022) 0,145	-0,004 (0,341) 0,027	-0,001 (0,801) 0,002
Slice 1	0,051 (0,051) 0,138	-0,024 (<0,001) 0,382	-0,011 (0,038) 0,156	-0,002 (0,695) 0,006
Slice 2	0,031 (0,143) 0,062	-0,014 (0,013) 0,167	-0,004 (0,421) 0,019	-0,0004 (0,928) 0,0002
Slice 3	0,023 (0,288) 0,033	-0,012 (0,026) 0,137	-0,003 (0,554) 0,010	-0,001 (0,877) 0,001
Slice 4	0,019 (0,386) 0,022	-0,012 (0,038) 0,120	-0,003 (0,554) 0,010	-0,001 (0,786) 0,002
Slice 5	0,020 (0,332) 0,028	-0,011 (0,046) 0,112	-0,004 (0,406) 0,020	-0,002 (0,661) 0,006
Slice 6	0,025 (0,224) 0,043	-0,011 (0,046) 0,112	-0,005 (0,288) 0,033	-0,002 (0,606) 0,008
Slice 7	0,033 (0,125) 0,068	-0,012 (0,031) 0,130	-0,005 (0,246) 0,039	-0,002 (0,681) 0,005
Slice 8	0,038 (0,079) 0,093	-0,012 (0,045) 0,120	-0,005 (0,240) 0,043	-0,002 (0,684) 0,005
Slice 9	0,041 (0,057) 0,120	-0,011 (0,063) 0,114	-0,007 (0,145) 0,072	-0,001 (0,825) 0,002

L1-L2

Multivariable

β (p)

	Pfirrmann	IVD Height	Tilt angle	Disc angle	R^2
Region 1	0,012 (0,692)	-0,007 (0,388)	0,0003 (0,963)	-0,008 (0,238)	0,1
Region 2	0,023 (0,420)	-0,011 (0,147)	0,001 (0,920)	-0,002 (0,718)	0,103
Region 3	0,029 (0,272)	-0,012 (0,084)	0,001 (0,893)	0,002 (0,642)	0,125
Region4	0,029 (0,231)	-0,013 (0,030)	0,001 (0,902)	0,005 (0,298)	0,184
Region 5	0,025 (0,244)	-0,016 (0,005)	0,00003 (0,995)	0,005 (0,218)	0,268
Mean	0,032 (0,157)	-0,013 (0,024)	-0,0004 (0,940)	0,002 (0,616)	0,211
Slice 1	0,028 (0,197)	-0,023 (<0,001)	-0,009 (0,079)	0,003 (0,483)	0,557
Slice 2	0,034 (0,125)	-0,015 (0,013)	0,0004 (0,940)	0,003 (0,511)	0,239
Slice 3	0,026 (0,265)	-0,013 (0,028)	0,001 (0,875)	0,002 (0,684)	0,175
Slice 4	0,020 (0,389)	-0,012 (0,046)	0,001 (0,922)	0,001 (0,813)	0,143
Slice 5	0,019 (0,397)	-0,011 (0,062)	-0,001 (0,923)	0,001 (0,879)	0,138
Slice 6	0,023 (0,31)	-0,011 (0,064)	-0,001 (0,806)	0,001 (0,854)	0,155
Slice 7	0,031 (0,173)	-0,012 (0,039)	-0,001 (0,794)	0,002 (0,692)	0,199
Slice 8	0,037 (0,116)	-0,012 (0,054)	-0,001 (0,820)	0,002 (0,656)	0,212
Slice 9	0,037 (0,109)	-0,010 (0,087)	-0,003 (0,532)	0,004 (0,439)	0,244

L2-L3

Univariable

β (p) R^2

	Pfirrmann	IVD Height	Tilt angle	Disc angle
Region 1	0,029 (0,235) 0,041	-0,006 (0,551) 0,011	0,004 (0,288) 0,033	-0,006 (0,393) 0,022
Region 2	0,030 (0,127) 0,067	-0,007 (0,404) 0,021	0,001 (0,634) 0,007	-0,006 (0,296) 0,032
Region 3	0,032 (0,032) 0,128	-0,006 (0,349) 0,026	-0,0003 (0,894) 0,001	-0,004 (0,388) 0,022
Region4	0,025 (0,063) 0,098	-0,008 (0,162) 0,057	-0,00006 (0,974) 0,00003	-0,002 (0,582) 0,009
Region 5	0,015 (0,275) 0,035	-0,011 (0,042) 0,117	0,0004 (0,845) 0,001	-0,003 (0,435) 0,018
Mean	0,025 (0,081) 0,087	-0,005 (0,367) 0,024	0,001 (0,539) 0,011	-0,003 (0,396) 0,021
Slice 1	0,003 (0,847) 0,001	-0,017 (0,022) 0,174	0,002 (0,287) 0,040	0,001 (0,786) 0,003
Slice 2	0,013 (0,395) 0,021	-0,009 (0,159) 0,058	0,003 (0,111) 0,073	0,001 (0,811) 0,002
Slice 3	0,017 (0,257) 0,038	-0,008 (0,175) 0,053	0,003 (0,209) 0,046	-0,001 (0,879) 0,001
Slice 4	0,022 (0,136) 0,064	-0,008 (0,216) 0,045	0,002 (0,411) 0,020	-0,002 (0,584) 0,009
Slice 5	0,025 (0,092) 0,081	-0,006 (0,315) 0,030	0,001 (0,673) 0,005	-0,004 (0,336) 0,027
Slice 6	0,028 (0,063) 0,098	-0,005 (0,409) 0,020	0,0002 (0,912) 0,0004	-0,005 (0,213) 0,045
Slice 7	0,030 (0,039) 0,119	-0,004 (0,522) 0,012	-0,00007 (0,973) 0,00004	-0,006 (0,136) 0,064
Slice 8	0,034 (0,034) 0,126	-0,003 (0,645) 0,006	-0,0003 (0,879) 0,001	-0,006 (0,171) 0,054

L2-L3

Multivariable

β (p)

	Pfirrmann	IVD Height	Tilt angle	Disc angle	R^2
Region 1	0,018 (0,482)	-0,003 (0,734)	0,007 (0,082)	-0,013 (0,144)	0,146
Region 2	0,022 (0,276)	-0,005 (0,555)	0,004 (0,277)	-0,008 (0,260)	0,129
Region 3	0,030 (0,060)	-0,005 (0,428)	0,0002 (0,944)	-0,001 (0,804)	0,15
Region4	0,024 (0,088)	-0,007 (0,192)	-0,00008 (0,972)	0,0004 (0,934)	0,148
Region 5	0,011 (0,415)	-0,011 (0,066)	0,001 (0,595)	-0,003 (0,596)	0,155
Mean	0,020 (0,177)	-0,004 (0,489)	0,003 (0,281)	-0,005 (0,359)	0,143
Slice 1	-0,001 (0,956)	-0,016 (0,040)	0,002 (0,550)	0,001 (0,922)	0,199
Slice 2	0,010 (0,508)	-0,008 (0,178)	0,004 (0,123)	-0,002 (0,695)	0,157
Slice 3	0,013 (0,383)	-0,008 (0,217)	0,003 (0,163)	-0,003 (0,557)	0,146
Slice 4	0,018 (0,241)	-0,007 (0,285)	0,003 (0,264)	-0,004 (0,486)	0,14
Slice 5	0,020 (0,196)	-0,005 (0,431)	0,002 (0,356)	-0,005 (0,361)	0,137
Slice 6	0,023 (0,153)	-0,004 (0,563)	0,002 (0,463)	-0,006 (0,306)	0,145
Slice 7	0,024 (0,114)	-0,002 (0,722)	0,002 (0,467)	-0,006 (0,225)	0,169
Slice 8	0,029 (0,090)	-0,001 (0,840)	0,001 (0,632)	-0,006 (0,331)	0,157

Slice 9	0,030 (0,067) 0,108	-0,001 (0,933) 0,0003	0,0002 (0,943) 0,0002	-0,006 (0,228) 0,048

Slice 9	0,024 (0,189)	0,001 (0,898)	0,002 (0,517)	-0,006 (0,336)	0,139

L3-L4

Univariable

β (p) R^2

	Pfirrmann	IVD Height	Tilt angle	Disc angle
Region 1	0,052 (0,023) 0,142	-0,002 (0,870) 0,001	0,003 (0,253) 0,038	-0,008 (0,226) 0,043
Region 2	0,046 (0,013) 0,167	0,001 (0,900) 0,0005	0,002 (0,291) 0,033	-0,008 (0,161) 0,057
Region 3	0,032 (0,033) 0,127	0,001 (0,848) 0,001	0,001 (0,443) 0,017	-0,007 (0,103) 0,076
Region4	0,009 (0,564) 0,010	0,001 (0,831) 0,001	-0,000063 (0,967) 0,00005	-0,009 (0,039) 0,120
Region 5	-0,012 (0,488) 0,014	0,004 (0,639) 0,007	-0,001 (0,376) 0,023	-0,012 (0,012) 0,171
Mean	0,020 (0,127) 0,067	0,002 (0,780) 0,002	0,001 (0,508) 0,013	-0,007 (0,082) 0,086
Slice 1	0,015 (0,294) 0,039	0,005 (0,491) 0,017	0,002 (0,189) 0,061	0,002 (0,607) 0,010
Slice 2	0,020 (0,115) 0,072	0,006 (0,354) 0,025	0,002 (0,142) 0,062	-0,001 (0,731) 0,004
Slice 3	0,024 (0,081) 0,087	0,004 (0,575) 0,009	0,001 (0,330) 0,028	-0,005 (0,237) 0,041
Slice 4	0,025 (0,083) 0,086	0,002 (0,722) 0,004	0,001 (0,418) 0,019	-0,007 (0,107) 0,075
Slice 5	0,027 (0,084) 0,085	0,001 (0,939) 0,0002	0,001 (0,452) 0,017	-0,008 (0,061) 0,100
Slice 6	0,024 (0,130) 0,066	-0,001 (0,938) 0,0002	0,001 (0,522) 0,012	-0,009 (0,038) 0,121
Slice 7	0,021 (0,177) 0,053	-0,002 (0,826) 0,001	0,001 (0,681) 0,005	-0,010(0,028) 0,134
Slice 8	0,016 (0,288) 0,033	-0,001 (0,889) 0,001	0,0002 (0,885) 0,001	-0,010(0,025) 0,139
Slice 9	0,009 (0,535) 0,011	0,00009 (0,989) 0,000006	-0,001 (0,701) 0,004	-0,010(0,021) 0,148

L3-L4

Multivariable

β (p)

	Pfirrmann	IVD Height	Tilt angle	Disc angle	R^2
Region 1	0,054 (0,016)	-0,003 (0,751)	0,005 (0,056)	-0,012 (0,072)	0,274
Region 2	0,048 (0,008)	0,0003 (0,972)	0,004 (0,060)	-0,011 (0,046)	0,308
Region 3	0,033 (0,023)	0,001 (0,893)	0,002 (0,120)	-0,009 (0,038)	0,265
Region4	0,009 (0,543)	0,001 (0,856)	0,001 (0,533)	-0,010 (0,038)	0,142
Region 5	-0,012 (0,430)	0,004 (0,603)	-0,001 (0,767)	-0,011 (0,025)	0,195
Mean	0,021 (0,101)	0,001 (0,842)	0,002 (0,171)	-0,008 (0,039)	0,206
Slice 1	0,016 (0,277)	0,004 (0,533)	0,002 (0,220)	-0,0002 (0,959)	0,119
Slice 2	0,022 (0,079)	0,005 (0,427)	0,002 (0,102)	-0,003 (0,398)	0,182
Slice 3	0,026 (0,060)	0,003 (0,631)	0,002 (0,138)	-0,007 (0,109)	0,201
Slice 4	0,027 (0,061)	0,002 (0,782)	0,002 (0,132)	-0,009 (0,044)	0,225
Slice 5	0,028 (0,061)	-0,0002 (0,974)	0,003 (0,110)	-0,011 (0,022)	0,249
Slice 6	0,025 (0,100)	-0,001 (0,838)	0,002 (0,121)	-0,012 (0,015)	0,245
Slice 7	0,021 (0,149)	-0,002 (0,741)	0,002 (0,180)	-0,012 (0,014)	0,231
Slice 8	0,016 (0,265)	-0,001 (0,837)	0,002 (0,316)	-0,011 (0,018)	0,197
Slice 9	0,009 (0,533)	0,0002 (0,975)	0,001 (0,713)	-0,010 (0,027)	0,162

L4-L5

Univariable

β (p) R^2

	Pfirrmann	IVD Height	Tilt angle	Disc angle
Region 1	0,035 (0,158) 0,058	0,014 (0,157) 0,058	0,002 (0,470) 0,015	0,009 (0,302) 0,031
Region 2	0,024 (0,256) 0,038	0,009 (0,271) 0,036	0,002 (0,314) 0,030	0,005 (0,507) 0,013
Region 3	0,013 (0,432) 0,018	0,005 (0,474) 0,015	0,001 (0,389) 0,022	0,001 (0,883) 0,001
Region4	0,011 (0,414) 0,020	0,002 (0,674) 0,005	0,001 (0,683) 0,005	-0,002 (0,678) 0,005
Region 5	0,011 (0,402) 0,021	0,003 (0,640) 0,007	-0,00007 (0,960) 0,00008	-0,003 (0,511) 0,013
Mean	0,016 (0,249) 0,039	0,006 (0,273) 0,035	0,001 (0,495) 0,014	0,001 (0,791) 0,002
Slice 1	-0,001 (0,930) 0,0003	0,002 (0,698) 0,005	0,001 (0,425) 0,023	0,001 (0,819) 0,002

L4-L5

Multivariable

β (p)

	Pfirrmann	IVD Height	Tilt angle	Disc angle	R^2
Region 1	0,086 (0,003)	0,019 (0,047)	0,003 (0,172)	0,020 (0,036)	0,317
Region 2	0,057 (0,023)	0,014 (0,116)	0,003 (0,137)	0,011 (0,166)	0,218
Region 3	0,027 (0,178)	0,007 (0,293)	0,002 (0,251)	0,004 (0,598)	0,097
Region4	0,015 (0,373)	0,005 (0,453)	0,001 (0,503)	-0,0004 (0,943)	0,048
Region 5	0,012 (0,482)	0,005 (0,441)	0,0003 (0,815)	-0,002 (0,745)	0,042
Mean	0,034 (0,051)	0,009 (0,125)	0,002 (0,255)	0,005 (0,377)	0,164
Slice 1	0,004 (0,838)	0,003 (0,623)	0,001 (0,416)	0,0004 (0,962)	0,033

Slice 2	0,007 (0,619) 0,007	0,004 (0,475) 0,015	0,001 (0,699) 0,004	0,003 (0,483) 0,015
Slice 3	0,014 (0,337) 0,027	0,006 (0,318) 0,029	0,001 (0,687) 0,005	0,003 (0,601) 0,008
Slice 4	0,021 (0,186) 0,051	0,007 (0,268) 0,036	0,001 (0,658) 0,006	0,002 (0,742) 0,003
Slice 5	0,022 (0,182) 0,052	0,008 (0,209) 0,046	0,001 (0,516) 0,013	0,002 (0,771) 0,003
Slice 6	0,021 (0,200) 0,048	0,008 (0,214) 0,045	0,001 (0,394) 0,021	0,001 (0,824) 0,001
Slice 7	0,020 (0,207) 0,046	0,007 (0,254) 0,038	0,001 (0,411) 0,020	0,001 (0,899) 0,0005
Slice 8	0,018 (0,264) 0,038	0,007 (0,249) 0,040	0,001 (0,527) 0,012	0,001 (0,812) 0,002
Slice 9	0,015 (0,311) 0,034	0,004 (0,515) 0,014	0,002 (0,331) 0,032	0,002 (0,751) 0,003

Slice 2	0,023 (0,217)	0,005 (0,413)	0,001 (0,569)	0,006 (0,306)	0,075
Slice 3	0,033 (0,076)	0,008 (0,200)	0,001 (0,448)	0,007 (0,277)	0,132
Slice 4	0,042 (0,031)	0,010 (0,120)	0,001 (0,352)	0,007 (0,278)	0,18
Slice 5	0,044 (0,025)	0,012 (0,077)	0,002 (0,232)	0,007 (0,302)	0,206
Slice 6	0,043 (0,029)	0,012 (0,074)	0,002 (0,162)	0,006 (0,367)	0,207
Slice 7	0,040 (0,040)	0,011 (0,096)	0,002 (0,182)	0,005 (0,439)	0,186
Slice 8	0,036 (0,064)	0,010 (0,120)	0,002 (0,281)	0,005 (0,431)	0,16
Slice 9	0,029 (0,113)	0,006 (0,314)	0,002 (0,252)	0,004 (0,534)	0,132

L5-S1

Univariable

β (p) R^2

	Pfirrmann	IVD Height	Tilt angle	Disc angle
Region 1	-0,015 (0,459) 0,016	0,012 (0,209) 0,046	0,002 (0,125) 0,041	0,006 (0,127) 0,067
Region 2	-0,009 (0,650) 0,006	0,002 (0,830) 0,001	0,003 (0,047) 0,111	0,006 (0,130) 0,066
Region 3	-0,008 (0,662) 0,006	-0,005 (0,576) 0,009	0,004 (0,013) 0,167	0,003 (0,383) 0,022
Region4	-0,009 (0,586) 0,009	-0,006 (0,410) 0,020	0,004 (0,003) 0,235	-0,001 (0,792) 0,002
Region 5	-0,006 (0,709) 0,004	-0,008 (0,284) 0,034	0,004 (0,001) 0,262	-0,001 (0,831) 0,001
Mean	-0,012 (0,437) 0,018	0,0002 (0,976) 0,00003	0,003 (0,010) 0,181	0,003 (0,290) 0,033
Slice 1	0,008 (0,642) 0,009	-0,003 (0,748) 0,004	0,003 (0,040) 0,164	0,002 (0,699) 0,006
Slice 2	-0,008 (0,653) 0,007	0,015 (0,046) 0,123	0,003 (0,023) 0,156	0,007 (0,043) 0,126
Slice 3	-0,009 (0,542) 0,011	0,008 (0,253) 0,038	0,003 (0,009) 0,185	0,004 (0,185) 0,051
Slice 4	-0,009 (0,547) 0,011	0,004 (0,584) 0,009	0,004 (0,007) 0,192	0,004 (0,229) 0,042
Slice 5	-0,012 (0,451) 0,017	0,001 (0,879) 0,001	0,004 (0,008) 0,191	0,004 (0,274) 0,035
Slice 6	-0,013 (0,431) 0,018	-0,002 (0,807) 0,002	0,003 (0,011) 0,175	0,003 (0,346) 0,026
Slice 7	-0,011 (0,518) 0,012	-0,005 (0,510) 0,013	0,003 (0,022) 0,145	0,003 (0,444) 0,017
Slice 8	-0,001 (0,970) 0,00005	-0,007 (0,422) 0,022	0,003 (0,109) 0,086	0,004 (0,336) 0,032
Slice 9	0,003 (0,895) 0,001	-0,006 (0,509) 0,018	0,003 (0,086) 0,113	0,004 (0,354) 0,034

L5-S1

Multivariable

β (p)

	Pfirrmann	IVD Height	Tilt angle	Disc angle	R^2
Region 1	-0,008 (0,698)	0,004 (0,734)	0,002 (0,341)	0,005 (0,312)	0,111
Region 2	-0,008 (0,682)	-0,009 (0,374)	0,003 (0,065)	0,007 (0,109)	0,184
Region 3	-0,010 (0,559)	-0,015 (0,123)	0,004 (0,016)	0,006 (0,168)	0,241
Region4	-0,010 (0,481)	-0,014 (0,104)	0,004 (0,003)	0,003 (0,412)	0,299
Region 5	-0,008 (0,601)	-0,013 (0,105)	0,004 (0,001)	0,001 (0,738)	0,33
Mean	-0,011 (0,459)	-0,008 (0,309)	0,003 (0,015)	0,004 (0,212)	0,233
Slice 1	0,006 (0,744)	-0,006 (0,580)	0,003 (0,051)	0,001 (0,757)	0,187
Slice 2	0,009 (0,601)	0,010 (0,238)	0,003 (0,062)	0,004 (0,306)	0,272
Slice 3	-0,002 (0,897)	0,003 (0,740)	0,003 (0,017)	0,003 (0,413)	0,224
Slice 4	-0,005 (0,734)	-0,003 (0,712)	0,003 (0,013)	0,004 (0,292)	0,224
Slice 5	-0,010 (0,508)	-0,008 (0,380)	0,003 (0,013)	0,004 (0,227)	0,236
Slice 6	-0,013 (0,392)	-0,011 (0,191)	0,003 (0,016)	0,005 (0,194)	0,239
Slice 7	-0,014 (0,389)	-0,015 (0,097)	0,003 (0,025)	0,005 (0,181)	0,23
Slice 8	-0,006 (0,761)	-0,016 (0,124)	0,003 (0,111)	0,006 (0,200)	0,184
Slice 9	-0,006 (0,830)	-0,015 (0,183)	0,003 (0,093)	0,005 (0,294)	0,201
